# Task Assignment and Path Planning for Multiple Autonomous Underwater Vehicles Using 3D Dubins Curves †

**DOI:** 10.3390/s17071607

**Published:** 2017-07-11

**Authors:** Wenyu Cai, Meiyan Zhang, Yahong Rosa Zheng

**Affiliations:** 1 School of Electronics & Information, Hangzhou Dianzi University, Hangzhou 310018, China; 2 School of Electrical Engineering, Zhejiang University of Water Resources and Electric Power, Hangzhou 310018, China; 3 Department of Electrical & Computer Engineering, Missouri University of Science and Technology, Rolla, MO 65409, USA; zhengyr@mst.edu

**Keywords:** target tracking, task assignment, multiple AUVs, energy balance, genetic algorithm

## Abstract

This paper investigates the task assignment and path planning problem for multiple AUVs in three dimensional (3D) underwater wireless sensor networks where nonholonomic motion constraints of underwater AUVs in 3D space are considered. The multi-target task assignment and path planning problem is modeled by the Multiple Traveling Sales Person (MTSP) problem and the Genetic Algorithm (GA) is used to solve the MTSP problem with Euclidean distance as the cost function and the Tour Hop Balance (THB) or Tour Length Balance (TLB) constraints as the stop criterion. The resulting tour sequences are mapped to 2D Dubins curves in the X−Y plane, and then interpolated linearly to obtain the *Z* coordinates. We demonstrate that the linear interpolation fails to achieve $G^{1}$ continuity in the 3D Dubins path for multiple targets. Therefore, the interpolated 3D Dubins curves are checked against the AUV dynamics constraint and the ones satisfying the constraint are accepted to finalize the 3D Dubins curve selection. Simulation results demonstrate that the integration of the 3D Dubins curve with the MTSP model is successful and effective for solving the 3D target assignment and path planning problem.

## 1. Introduction

Autonomous Underwater Vehicles (AUVs) and underwater gliders have found important applications in ocean exploration, oil and gas production, environmental monitoring, underwater infrastructure monitoring, weather services, and coastal surveillance [1,2,3]. Typically, these vehicles are programmed to visit a number of predetermined targets, perform some tasks at the target locations, and then return home. With the increased demand and commercial success of the AUVs and gliders, it is of increasing interests to employ a fleet of vehicles simultaneously and cooperatively to perform a mission. Therefore, multi-vehicle task assignment and path planning become an important research topic in recent years.

Due to the size, weight, and fuel constraints, these vehicles have strong limitations in underwater missions, such as limited mission length, stringent nonholonomic motion constraints, and limited communication with each other or with the home base. A nonholonomic motion constraint requires that the vehicle motion is along a smooth curvature without reversing direction. This often requires that the vehicle paths satisfy geometric continuity to support their kinematic constraints [4,5]. For point-to-point path planning, Dubins curves have been widely utilized to achieve $G^{1}$ continuity and shortest path length [6,7]. Recent literatures on Dubins vehicles also consider environmental conditions such as ocean currents [8,9], obstacle avoidance [10,11], and vehicle/glider characteristics [12,13]. However, most of the works only consider 2-dimensional (2D) Dubins curve, and the extension to 3D Dubins curve is recently proposed in [14] for unmanned aerial vehicles by using linear interpolation. This method is also adopted in [12,13] for path planning of gliders and AUVs.

The multi-target multi-AUV task assignment and path planning problem is commonly modeled by the multiple traveling salesperson (MTSP) problem. In the review paper [10], Zhu et al. provided a detailed report on the recent progress in this area. The MTSP problems are often solved by the K-means clustering method [8], the genetic algorithm (GA) [15,16], or the heuristic search algorithms [17,18]. Due to the high computational complexity, the MTSP problem is often solved by using the Euclidean distances between targets as the cost function. The resulting task assignments and tour sequences are then post-processed to account for vehicle dynamics, environmental constraints, and possible environmental changes. Ocean environmental conditions, such as the effect of strong ocean current, are considered in many recent works [8,9,17,19,20]. In addition, several approaches have been developed to adapt to changing environment, including the fast marching-based approach in [20], the Self Organizing Map (SOM) neural network approach in [21], and the dynamic task assignment approach in [22].

To account for the nonholonomic motion constraint, the tour sequence is mapped to point-to-point Dubins curves with a constraint that the incoming and outgoing headings at the joints are the same. In a large tour sequence where the number of targets to be visited is large, the search for shortest Dubins path is also computationally intensive. Several approaches have been proposed in the literature. An Alternating Algorithm is proposed in [23], which only maps half of the tour points to Dubins curves, thus reducing the search size for the shortest Dubins path. Two beading methods have also been presented in [23] to map the point-to-point paths with shortest bead-shaped paths. Other path-smoothing methods are presented in [24], which use continuous-curvature paths such as Clothoids, Bezier curves, and B-splines.

Most of the Dubins TSP solutions also have the limitation of using the 2D Dubins curves without considering the 3D underwater space. A few recent works extend the 2D Dubins curves to 3D [12,13,14] without considering multiple targets. Some works in 3D multi-target task assignment [11] consider targets in the 3D space without path smoothing. In this paper, we integrate the 3D Dubins curve with the MTSP model for 3D multi-target task assignment and path planning. We impose the energy consumption constraint of each AUV with the Tour Hop Balance (THB) and Tour Length Balance (TLB) criteria in the GA algorithm when solving for the tour sequences of multiple AUVs. We call these algorithms the THB-3Dubins-MTSP algorithm and TLB-3Dubins-MTSP algorithm, respectively. We investigate the simple linear interpolation method of 3D Dubins curve in the multi-target path planning scenario and demonstrate that the simple 3D Dubins curves fail to meet with $G^{1}$ continuity at multiple targets, because although the 3D Dubins path may be $G^{1}$ continuous in the X−Y plane, they are discontinuous in the *Z*-domain.

Based on this finding, we propose a simple solution for accommodating the vehicle dynamics. The interpolated 3D Dubins curves are checked against the AUV dynamics constraint in the *Z*-domain and the ones satisfying the constraint are accepted to finalize the 3D Dubins curve selection. We call this rejection-acceptance method. Simulation results demonstrate that the integration of the 3D Dubins curve with the MTSP model is successful and effective for solving the 3D target assignment and path planning problem.

## 2. Problem Statement

**Figure 1:**
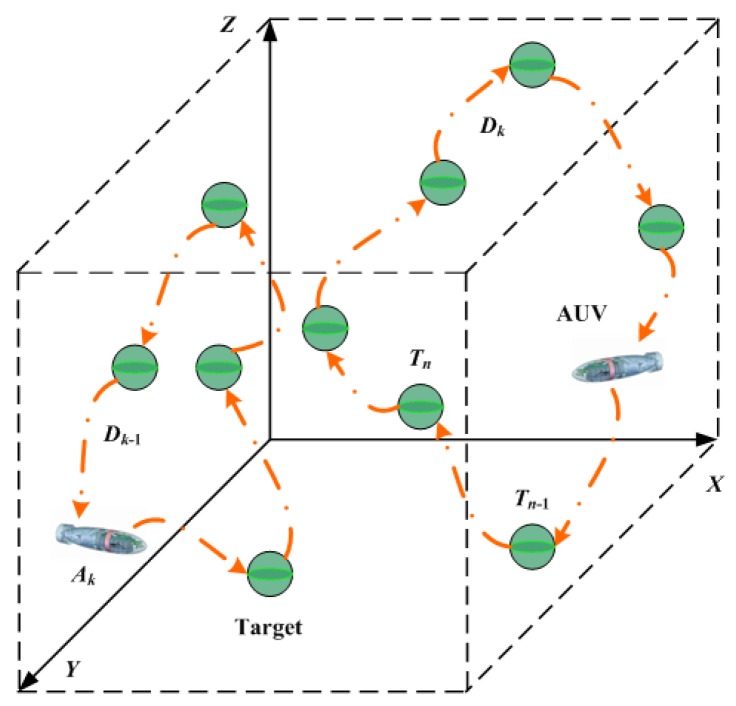
Targets tracking with multiple AUVs.

Consider multiple AUVs constituting a collaborative team and performing the mission of tracking multiple underwater targets in a 3D underwater environment, as shown in Figure 1. Assume a set of static targets $T=\{T_{1},T_{2},\ldots ,T_{N}\}$ and a set of homogeneous mobile AUVs $A=\{A_{1},A_{2},\ldots ,A_{K}\}$ that are randomly deployed in the X×Y×Z three dimensional underwater space, with *N* and *K* denoting the total numbers of static targets and mobile AUVs, respectively. Also assume K<N, as this is commonly encountered in many practical applications. Each AUV has the same initial energy $E_{init}$ and the same energy consumption model which is a linear function of its tour length. In order to illustrate design detailed methodology of proposed algorithm, we summarize the simplified notation in Table 1 for the reader’s convenience.

The objective of task assignment and path planning is to assign a tour sequence of targets $D_{k},k=1,2,\ldots ,K$ from the target set T to each AUV such that each target is visited by an AUV once and only once, and the total cost of visiting all targets is minimized.

Let $N_{k}$ and $L_{k}$ denote the number of targets and tour length of sequence $D_{k}$, respectively. The tour cost of tour sequence $D_{k}$ is denoted as $C(D_{k})$, and the task assignment and path planning problem is to minimize

(1) $$C_{Total}=\sum_{k=1}^{K}C(D_{k})$$

subject to

(2) $$\begin{array}{c} T=\overset{K}{\underset{k=1}{⋃}}D_{k} \end{array}$$

(3) $$\begin{array}{c} D_{k}⋂D_{l}=\emptyset ,\;\forall k,l\;\mathrm{and}\;k\neq l \end{array}$$

(4) $$\begin{array}{c} \mathrm{Var}(N_{k})\to 0 \end{array}$$

(5) $$\begin{array}{c} \mathrm{Var}(L_{k})\to 0 \end{array}$$

where Equations (4) and (5) are the Tour Hop Balance (THB) constraint and the Tour Length Balance (TLB) constraint, respectively, and Var(·) denotes the intra-AUV variance which is calculated in the following section, → means as small as possible. For example, $N_{k}=\frac{N}{K},\forall k$ if *N* is divisible by *K*.

### 2.1. Vehicle Kinematic Constraints

**Figure 2:**
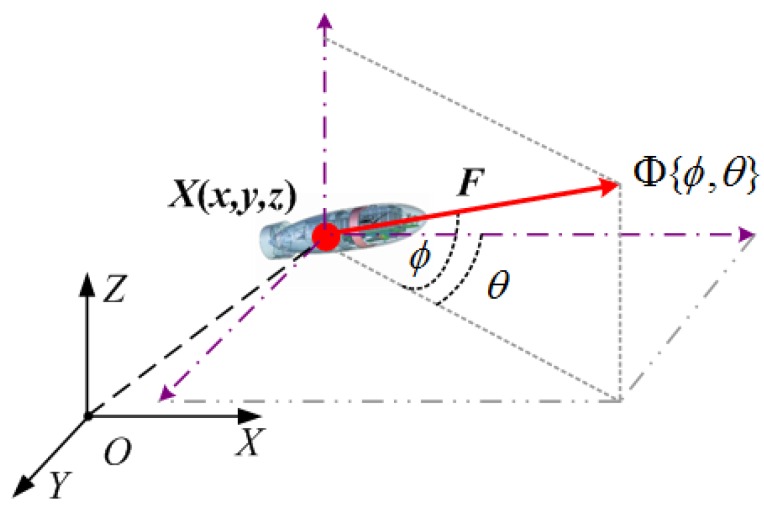
AUV and its motion heading in 3D Cartesian space.

An AUV belongs to a body-fixed coordinate system with six degrees of freedom, and so its motion can be described relative to an inertial-fixed reference frame. However, we only consider the position value and motion heading of an AUV in this paper since it is enough for path planning with Z-axis linear interpolation method. The location and motion of an AUV in three-dimensional Cartesian space are shown in Figure 2, where the position of the AUV is denoted as X={x,y,z}, and its motion heading is denoted as Φ={ϕ,θ}, where θ and ϕ are the X-Y plane angle and Z-axis angle projected from the AUV’s motion heading, respectively. The velocity scalar of the AUV is denoted as ***F***, and the projected X-Y plane angle and Z-axis angle are bounded, making its motion nonholonomic constraints [8] such that

(6) $$\left\{\begin{array}{c} \dot{x}=F\cos\theta \cos\phi \\ \dot{y}=F\cos\theta \sin\phi \\ \dot{z}=F\sin\theta \end{array}\right.$$

where the dot operator is the derivative, and

(7) $$\begin{array}{c} \dot{\phi }=\xi ,\;\xi \in \left[-\omega_{1},\omega_{1}\right] \end{array}$$

(8) $$\begin{array}{c} \dot{\theta }=\Theta ,\;\Theta \in \left[-\omega_{2},\omega_{2}\right] \end{array}$$

where $\omega_{1}$ and $\omega_{2}$ represent the curvature bounds. The nonholonomic constraints requires that the AUV paths satisfy the $G^{0}$ and $G^{1}$ continuities [24,25] which are defined as follows:

$G^{0}$ *continuity*: P(u)=[x(u),y(u),z(u)] and Q(w)=[x(w),y(w),z(w)] be two regular parametric 3D curves in the X×Y×Z space, where u∈[0,1] and w∈[0,1] are the parameters with 0 and 1 denoting the starting and ending points, respectively. If P(1)=Q(0)=J, then the two parametric curves meet at joint *J* with $G^{0}$ continuity.

$G^{1}$ *continuity*: If $\dot{P}\left(u\right){{|}_{u=1}=\dot{Q}\left(w\right)|}_{w=0}$, then the two parametric curves meet at joint *J* with $G^{1}$ continuity.

### 2.2. The 2D Dubins Curve

**Figure 3:**
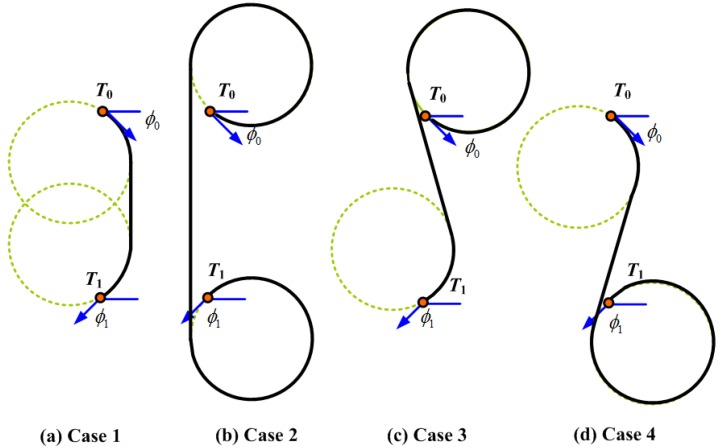
Four CSC types of 2D Dubins curves with $\phi_{0}=-\pi /4$ and $\phi_{1}=-3\pi /4$.

For 2D path planning kinematic constraints, a classical path model is to use the 2D Dubins curve [6,7] to satisfy the $G^{1}$ continuity. Given any two points in the X×Y plane, starting at $x_{0}=\left(x_{0},y_{0}\right)$ and ending at $x_{1}=\left(x_{1},y_{1}\right)$, the Dubins curves satisfy the dynamic constraints expressed in Equations (6) and (7) by a combination of maximum curvature arcs (C) and/or a straight line segment (S) to form two families of curves: family CCC and family CSC. Note that all arcs are with radius ρ. The family CCC contains curves with types RLR and LRL, where *R* and *L* denote a right turn arc (or clockwise) and a left turn arc (counter clock), respectively; The family CSC includes four types: RSR, LSL, RSL, and LSR, where *S* is a straight line segment. Therefore, the shortest Dubins path between two points are selected from the six types ℜ={LSL,RSR,RSL,LSR,RLR,LRL}. For example, Figure 3 shows the four CSC types of Dubins curves with $\phi_{0}=-\pi /4$ and $\phi_{1}=-3\pi /4$ and two points (0,0) and (0,−d). Note that $\phi_{0}$ and $\phi_{1}$ are measured counter-clockwise with respect to the positive x-axis. It is obvious that the Type 1 Dubins curve has the shortest length.

The 2D Dubins curves have been well investigated in the literature. It has been shown [7] that for the long path case where the distance between the starting point and ending point, denoted as *d* and normalized by the turning radius ρ, satisfies $d>\sqrt{4-(|\cos\phi_{0}|+|\cos\phi_{1}{\left|\right)}^{2}}+|\sin\phi_{0}|+|\sin\phi_{1}|$, the shortest path cannot be in the CCC family. The shortest paths with given $\phi_{0}$ and $\phi_{1}$ can be easily determined by the quadrants that the two angles fall in Ref. [7]. The elements of the 4×4 matrix, $a_{i,j}$, represents the quadrant number *i* of the starting angle and the quadrant number *j* of the ending angle. The shortest 2D Dubins curves starting from (0,0) and ending at (d,0) are then determined by Table 2, where the different types in Table 2 are determined by certain switching functions defined in [7].

The exact path and its length can be calculated by the three operators, $L_{\gamma }$ for left turn, $R_{\gamma }$ for right turn, and $S_{\gamma }$ for straight, which transform an arbitrary point [x,ϕ] into its corresponding image point

(9) $$\left\{\begin{array}{c} L_{\gamma }\left(x,\phi \right)=\left[\left(x+\sin\left(\phi +\gamma \right)-\sin\phi ,y-\cos\left(\phi +\gamma \right)+\cos\phi \right),\phi +\gamma \right]\\ R_{\gamma }\left(x,\phi \right)=\left[\left(x-\sin\left(\phi -\gamma \right)+\sin\phi ,y+\cos\left(\phi -\gamma \right)-\cos\phi \right),\phi -\gamma \right]\\ S_{\gamma }=\left[\left(x+\gamma \cos\phi ,y+\gamma \sin\phi \right),\phi \right] \end{array}\right.$$

where x=(x,y), and the index γ means that the path segment is of length γ. For example, the LSR path with respective lengths of t,p,q between point $\left[\left(0,0\right),\phi_{0}\right]$ and $\left[\left(d,0\right),\phi_{1}\right]$ is solved by

(10) $$R_{q}\left(S_{p}\left(L_{t}\left(\left[\left(0,0\right),\phi_{0}\right]\right)\right)\right)=\left[\left(d,0\right),\phi_{1}\right]$$

or

(11) $$\left\{\begin{array}{c} p\cos\left(\phi_{0}+t\right)+2\sin\left(\phi_{0}+t\right)-\sin\phi_{0}-\sin\phi_{1}=d,\\ p\sin\left(\phi_{0}+t\right)-2\cos\left(\phi_{0}+t\right)-\cos\phi_{0}-\cos\phi_{1}=0,\\ \phi_{0}+t-q=\phi_{1}\left\{\mathrm{mod}2\pi \right\} \end{array}\right.$$

The solution is denoted $t_{LSR}$, $p_{LSR}$, and $q_{LSR}$, respectively. The total path length is then $L_{LSR}=t_{LSR}+p_{LSR}+q_{LSR}=\phi_{1}-\phi_{2}+2t_{LSR}+p_{LSR}$. Details of other types of paths can be found in [7].

### 2.3. The Multiple Traveling Salesmen Problem for Dubins Vehicle

Multi-vehicle path planning is often casted as a Multiple Traveling Salesmen Problem (MTSP) [15] which is to find a set of closed paths for multiple traveling salesmen to visit a set of cities such that each and every city is visited exactly once and the total cost of visiting all cities are minimized. In the AUV path planning problem, the cost is the sum of Euclidean distances along the paths. It is difficult to find the optimal solution to the MTSP and heuristic iterative algorithms, such as the Genetic Algorithm (GA), Reactive Tabu Search, and Clustering and Actioning [8] are often used. In this work, we use the GA algorithm [15].

The MTSP is first converted into an equivalent mixed integer programming (MIP) problem [15] by introducing a binary selection variable $I_{i,j}^{k}\in \left\{0,1\right\}$ defined as the indicator for the *k*th iteration of GA algorithm. If $I_{i,j}^{k}=1$, then the AUV $A_{k}$ is assigned to travel from target $T_{i}$ to target $T_{j}$. Otherwise, if $I_{i,j}^{k}=0$, then AUV $A_{k}$ is assigned not to visit targets $T_{i}$ and $T_{j}$. Given an undirected graph G=(T,E), where T is the set of static targets (nodes), E={(i,j),i,j∈T} is the set of edges, and i,j=0,1,…,N with i=0 and j=0 denoting the home node or the starting/returning location of the AUVs. A cost matrix $C=\{c_{i,j}\}$ is defined on edges (i,j) associated with E. The MIP optimization is to

(12) $$\mathrm{minimize}\;\sum_{i=0}^{N}\sum_{j=0}^{N}\sum_{k=1}^{K}c_{i,j}I_{i,j}^{k}$$

subject to

(13) $$\begin{array}{c} \sum_{j=1}^{N}\sum_{k=1}^{K}I_{1,j}^{k}=K, \end{array}$$

(14) $$\begin{array}{c} \sum_{i=1}^{N}\sum_{k=1}^{K}I_{i,1}^{k}=K, \end{array}$$

(15) $$\begin{array}{c} \sum_{j=0}^{N}\sum_{k=1}^{K}I_{i,j}^{k}=1,\;i=2,3,\ldots ,N. \end{array}$$

(16) $$\begin{array}{c} \sum_{i=0}^{N}\sum_{k=1}^{K}I_{i,j}^{k}=1,\;j=2,3,\ldots ,N. \end{array}$$

(17) $$\begin{array}{c} \sum_{i}\sum_{j}c_{i,j}I_{i,j}^{k}\leq S,\;k=1,2,\ldots ,K. \end{array}$$

The constraints (13) and (14) ensure that the *K* salesmen start from the home node and return to the home node. Constraints (15) and (16) ensure that each node is visited (entered and left) only once. The constraint (17) is to ensure that the cost of each AUV is capped at *S*. Note that the constraints in (4) and (5) are used as the stop criterion.

The GA algorithm solves the MTSP by treating all possible tour sequences as the population, a specific tour sequence as an individual, the nodes in a tour sequence as a chromosome, and the travel length of a tour sequence as the fitness function. The GA algorithm starts with a random population with *M* individuals, and calculates the fitness function for each of the *M* individuals; then it creates new population by parent selection, parent crossover, chromosome mutation, and descendant acceptance; A new population results from replacing individuals by descendants with better fitness. Next the generated new population is used in the next iteration that iterates through the new population generation process, until the stop condition is satisfied. The solution to the MTSP is the set of selection variables $I_{i,j}^{k}$. The tour sequence for the *k*th AUV is the set of edges selected by the GA algorithm with $I_{i,j}^{k}=1$. That is

(18) $$D_{k}=\left\{\left(i,j\right)|I_{i,j}^{k}=1\right\}\mapsto \left\{T_{n}\right\},\;k=1,\ldots ,K.$$

Note that the AUV dynamics and $G^{1}$ continuity constraints have to be considered when applying the MTSP model to AUVs target assignment and path planning. Using Dubins curves, the MTSP model can be applied to solve the Dubins target assignment and path planning problem in three steps:

- Step 1 uses the Euclidean distances between the nodes as the cost $c_{i,j}$ and assigns the *N* targets to the *K* AUVs through the GA algorithm.
- Step 2 converts the tour sequence of each AUV into the Dubins paths by selecting a set of headings at each node and computing the lengths of Dubins curves;
- Step 3 chooses the Dubins path and its associated headings with the shortest total distance.

The headings of the AUVs are discretized to $2^{B}$ angles such that $\phi_{0},\phi_{1},\theta_{0}$ and $\theta_{1}$ take values at $\pi b/2^{B}$ with $b=1,\ldots ,2^{B+1}-1$ and excluding multiples of π/2. The discretization can greatly reduce the computational complexity in searching for the shortest Dubins path. The total length of a tour sequence is then calculated as

(19) $$C\left(D_{k}\right)=L\left(\left[X_{N_{k}},\Phi_{N_{k}}\right];\left[X_{0},\Phi_{0}\right]\right)+\sum_{m=0}^{N_{k}-1}L\left(\left[X_{m},\Phi_{m}\right];\left[X_{m+1},\Phi_{m+1}\right]\right)$$

for k=1,…,K, and the total cost of all AUVs is computed as in (1).

In comparison, an existing method called the Alternating Algorithm [23] also uses the first approach that solves the Euclidean MTSP then maps the tour sequences to Dubins path. However, to reduce the computational complexity of the Dubins search, only odd-indexed edges in the tour sequence are replaced by minimum length Dubins paths, the even-indexed edges keep the straight Euclidean path.

## 3. Target Assignment and Path Planning in 3D Space

This section extends the target assignment and path planning algorithms from 2D to 3D by incorporating the 3D Dubins curves. We use the first approach in which the GA algorithm solves the Euclidean MTSP for the multiple targets and multiple AUVs, then maps the Dubins curves in 3D. We follow the simple linear interpolation method in [13,14] and analyze the $G^{1}$ continuity of the resulting 3D Dubins paths.

### 3.1. The 3D Dubins Curves and Their Path Lengths

**Figure 4:**
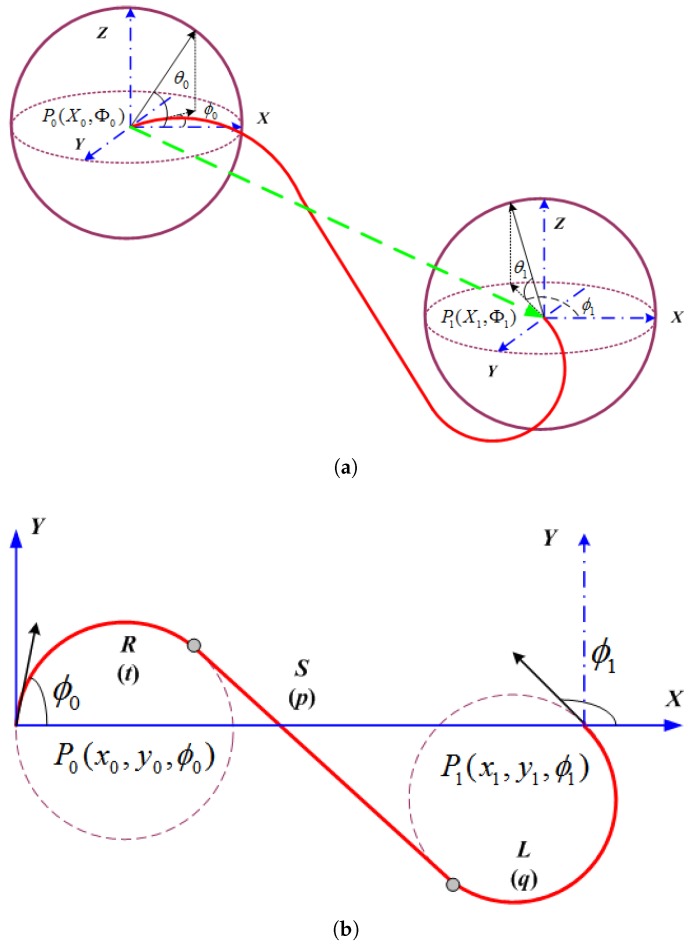
The RSL example of 2D and 3D Dubins curves. (**a**) 3D coordinates and its interpolated 3D Dubins curve; (**b**) 2D Dubins curve.

To extend the 2D Dubins curves to 3D space using the linear interpolation method, the 3D tour sequences are first projected on to the 2D X×Y plane in a global coordinate system. Taking a starting point $\left[X_{0},\Phi_{0}\right]$ and an ending point $\left[X_{1},\Phi_{1}\right]$ in the 3D space and project them on to the 2D plane, as shown in Figure 4. Then the starting and ending points become 2D parameters $\left[\left(x_{0},y_{0}\right),\phi_{0}\right]$ and $\left[\left(x_{1},y_{1}\right),\phi_{1}\right]$. The 2D Dubins curve is designed as described in Section 2.2, and the lengths of the arcs and line segment are calculated by (10). Let $L_{0,x}$ and $L_{0,1}$ denote the lengths along the 2D Dubins curve from $\left(x_{0},y_{0}\right)$ to (x,y) and from (x,y) to $\left(x_{1},y_{1}\right)$, respectively.

The linear interpolation adds the *z* coordinate by

(20) $$z=z_{0}+\frac{L_{0,x}}{L_{0,1}}\left(z_{1}-z_{0}\right)$$

where $z_{0}$ and $z_{1}$ are the *Z* coordinates of the starting and ending points. The resulting 3D Dubins curve is illustrated in Figure 4a.

The length of the interpolated 3D Dubins curve is calculated as

(21) $$\begin{array}{c} L_{3D}=\sqrt{t^{2}+{\left(z_{0}-z_{m}\right)}^{2}}+\sqrt{p^{2}+{\left(z_{m}-z_{n}\right)}^{2}}+\sqrt{q^{2}+{\left(z_{n}-z_{1}\right)}^{2}} \end{array}$$

where t,p, and *q* are the CSC segment lengths of the 2D Dubins curve, and $z_{m}$ and $z_{n}$ are the *Z* coordinates of the segment joints which are linearly interpolated as

(22) $$\begin{array}{ccc} z_{m} & = & z_{0}+\frac{t}{t+p+q}\left(z_{1}-z_{0}\right), \end{array}$$

(23) $$\begin{array}{ccc} z_{n} & = & z_{0}+\frac{t+p}{t+p+q}\left(z_{1}-z_{0}\right)=z_{1}-\frac{q}{t+p+q}\left(z_{1}-z_{0}\right). \end{array}$$

**Figure 5:**
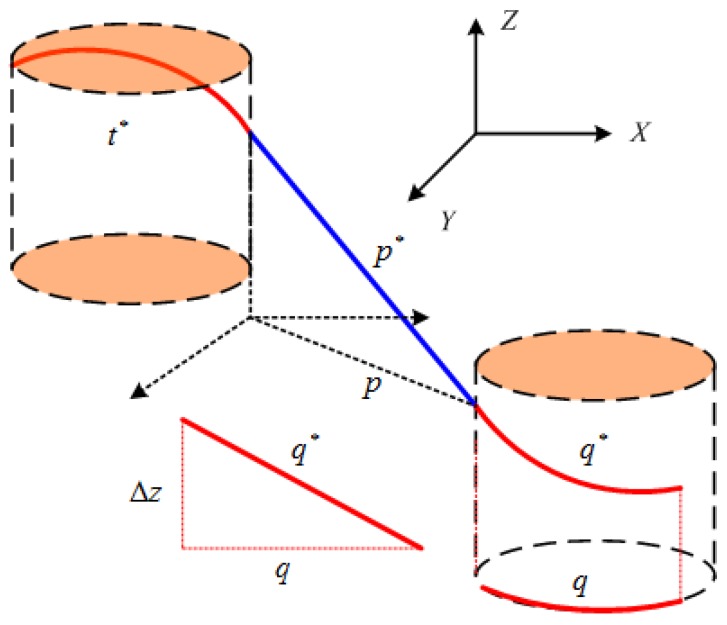
3D cylindrical spiral model.

Equation (21) is easily derived from Figure 5, since for the straight segment, the sides with length $p,p^{*}$, and $z_{m}-z_{n}$ form a right triangle. Thus, $p^{*}=\sqrt{p^{2}+{\left(z_{m}-z_{n}\right)}^{2}}$. For the left turn segment (the ending segment in this example), $q^{*}=\sqrt{q^{2}+{\left(z_{1}-z_{n}\right)}^{2}}$ if the cylinder containing the arc segments $q^{*}$ and *q* is opened and laid flat, thus the segments $q,q^{*}$ and $z_{m}-z_{1}$ form a right triangle. Similar to the left turn segment, the right turn segment satisfies $t^{*}=\sqrt{t^{2}+{\left(z_{m}-z_{0}\right)}^{2}}$. As a result, the total length of the 3D Dubins curve is $L_{3D}=t^{*}+p^{*}+q^{*}$.

Next, we show that the shortest 2D Dubins curve results in the shortest 3D Dubins curve and present the proof in Theorem 1. We also analyze the $G^{1}$ continuity of the interpolated 3D Dubins curves and present the results in Theorem 2.

**Theorem** **1.** The shortest 2D Dubins curve corresponds to the shortest 3D Dubins curve if linear interpolation is used to generate the Z coordinates.

**Proof.**  Let $L_{2D}=t+p+q$ be the length of the 2D Dubins curve. Substituting (22) into (21) yields

(24) $$\begin{array}{cc} L_{3D} & =t\sqrt{1+\frac{{\left(z_{1}-z_{0}\right)}^{2}}{L_{2D}^{2}}}+p\sqrt{1+\frac{{\left(z_{1}-z_{0}\right)}^{2}}{L_{2D}^{2}}}+q\sqrt{1+\frac{{\left(z_{1}-z_{0}\right)}^{2}}{L_{2D}^{2}}}\\ & =\left(t+p+q\right)\times \sqrt{1+\frac{{\left(z_{1}-z_{0}\right)}^{2}}{L_{2D}^{2}}}\\ & =\sqrt{L_{2D}^{2}+{\left(z_{1}-z_{0}\right)}^{2}}\\ & \leq L_{2D}+\left|z_{1}-z_{0}\right| \end{array}$$

Therefore, the shortest length $L_{2D}$ of 2D Dubins curve leads to the shortest 3D Dubins curve. ☐

**Theorem** **2.** The 3D Dubins curves generated by linear interpolation of Z coordinates from the 2D Dubins curve can preserve $G^{1}$ continuity in the X−Y plane but would lose the $G^{1}$ continuity in Z.

**Proof.**  As shown in Figure 4, the 2D Dubins curve between a starting and an ending target is composed of three segments: arc, line, and arc, which joint at two joints. These three segments meet at the joints with $G^{1}$ continuity because the line segment designed by (10) ensures the $G^{1}$ continuity of each Dubins curve on the X−Y plane. When the tour sequence contains multiple targets, the two Dubins curves meet at one target location, and the $G^{1}$ continuity can be preserved by forcing the end heading $\phi_{1}$ of the first Dubins curve equal to the start heading $\phi_{0}$ of the second Durbins curve.

However, in the *Z* domain, the linear interpolation among three or more targets cannot guarantee the $G^{1}$ continuity at the joints. For example, let targets 1, 2, and 3 be linked by two line segments. The end of the first line segment joins the start of the second line segment. when the two line segments are not aligned, the start heading $\theta_{0}$ of the second line segment (in *Z* domain) is not equal to the end heading $\theta_{1}$ of the first line segment. Therefore, the two line segments meet at the joint without $G^{1}$ continuity. For a particular example: $z_{i-1}=10$, $z_{i}=15$, $z_{i+1}=10$, so the AUV will have to increase its height from X(i−1) to X(i), and it must decrease its height from X(i) to X(i+1) . If the AUV has an initial upward heading angle $\phi_{i-1}$ because $z_{i}-z_{i-1}=5$, then it might turn to a downward heading $\phi_{i}$ at X(i) because $z_{i+1}-z_{i}=-5$. Therefore, the AUV will have to change its heading angle Φ=(ϕ,θ) suddenly before traveling down to $z_{i+1}=10$. ☐

### 3.2. Path Planning for Multiple AUVs in 3D Space

This section describes the detailed solution to the multiple targets tracking task assignment problem in three dimensional underwater workspace with constraints of Tour Hop Balance (THB) or Tour Length Balance (TLB). The three step approach in Section 2.3 is used, where step 1 applies the multiple traveling salesman problem (MTSP) algorithm with Euclidean distances as the fitness function.

(25) $$\mathrm{fitness}=\frac{1}{\sum_{k}L_{e}\left(X_{D_{k}}\right)}$$

To incorporate the Tour Hop Balance (THB) or Tour Length Balance (TLB) constraints into the Genetic Algorithm, the variances of $N_{k}$ and $L_{k}$ are estimated as

(26) $$\begin{array}{cc} {\hat{\sigma }}_{N_{k}} & =\frac{1}{K-1}\sum_{k=1}^{K}{\left(N_{k}-\hat{\mu }\left(N_{k}\right)\right)}^{2} \end{array}$$

(27) $$\begin{array}{cc} {\hat{\sigma }}_{L_{k}} & =\frac{1}{K-1}\sum_{k=1}^{K}{\left(L_{k}-\hat{\mu }\left(L_{k}\right)\right)}^{2} \end{array}$$

where $\hat{\mu }\left(N_{k}\right)$ and $\hat{\mu }\left(L_{k}\right)$ are the estimated expectations of $N_{k}$ and $L_{k}$, respectively. These variances are used by the GA as the termination rule. The resulting algorithms are called the THB-3Dubins-MTSP algorithm and TLB-3Dubins-MTSP algorithm, respectively. The outputs of the GA algorithms are a set of tour sequences for all AUVs.

Then step 2 maps the direct paths in a tour sequence into 2D Dubins curves, which is accomplished by projecting the 3D target locations onto the X−Y plane and design 2D Dubins curves. Since different headings on the 2D plane can result in different solutions, the curves include all possible starting $\phi_{0}$ and ending $\phi_{1}$ for all target pairs. The shortest 2D path is selected by computing the total distances and selecting the smallest one.

The last step converts the 2D Dubins path into 3D curves by linear interpolation of the *Z* coordinates at each target pair. Then the headings $\theta_{0}$ and $\theta_{1}$ are calculated. Since linear interpolation loses the $G^{1}$ continuity, the difference between $\theta_{0}$ and $\theta_{1}$ at each joint *J* of the 3D Dubins path is computed as $\Theta \left(J\right)=\theta_{1}\left(J\right)-\theta_{0}\left(J\right)$. The results Θ(J) for all *J* are compared against the bound $\omega_{2}$. If any joint of the Dubins path has a $\Theta \left(J\right)>\omega_{2}$, then the 3D Dubins path is rejected, and another 2D Dubins path, computed in Step 2, has to be used to be interpolated to the 3D Dubins curve. The process is repeated until the 3D Dubins path satisfies the vehicle dynamics constraint. This method is called rejection-acceptance method.

In summary, the pseudo-code of the proposed algorithms is described in Algorithm 1. For all we know, there are a small collision probabilities when multiple robots cruising in the same 3D underwater space. Two and more than two AUVs will be collided when multiple AUVs are arriving at the same coordinates at the same time. However, we run simulations of MTSP for collision check and find the collision between AUVs is negligible since the quantity of AUVs is small because of its high cost. Moreover, another sub-optimal paths can be generated with GA if there is a collision.

**Algorithm 1** The pseudo-code of the proposed algorithms**Input:**
Set of static targets T;
Set of mobile AUVs A;
Total number of static targets *N*;
Total number of mobile AUVs *K*;**Output:**
Tour sequence for the *k*th AUV: $D_{k}$, k=1,2,…,K;
Tour trajectory for the *k*th AUV: $S_{k}$, k=1,2,…,K;1:Initialization: n=1;2:**while**
(n≤N)
**do**3: if(THB-3Dubins-MTSP)4:  # GA based MTSP calculation5:  # Break when reaching THB constraint;6: else if (TLB-3Dubins-MTSP)7:  #GA based MTSP calculation8:  Break when reaching TLB constraint;9: end if10: n:=n+1;11:**end while**12:Initialization: k=1, $S_{k}:=\emptyset$;13:**while**
(k≤K)
**do**14: Initialization: v=0;15: **while**
$\left(v<N_{k}\right)$
**do**16:  # 3D Dubins curve plotting17:  $S_{k}:=\left[S_{k},3D\_\mathrm{Dubins}\left(T_{v},T_{v+1}\right)\right]$;18:  v:=v+1;19: **end while**20: $S_{k}:=\left[S_{k},3D\_\mathrm{Dubins}\left(T_{N_{k}},T_{0}\right)\right]$;21: k:=k+1;22:**end while**23:**return**$S_{k}$, for k=1,2,…,K

## 4. Simulation Results

**Figure 6:**
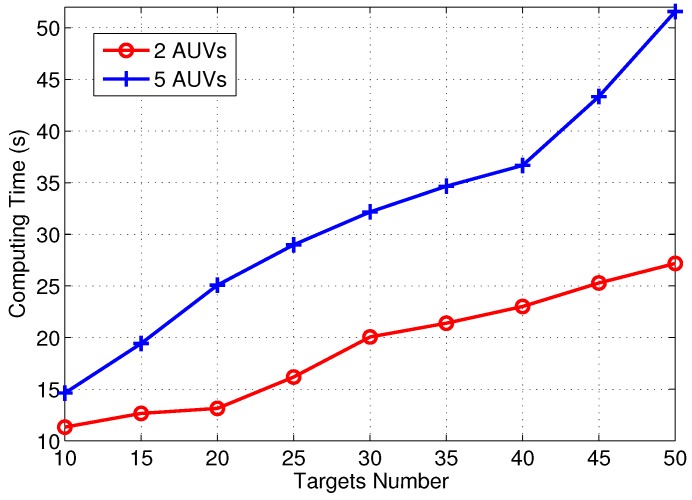
Computing time comparison.

Simulations were set up with multiple underwater targets deployed randomly in a cube of 20×20×10 units, where 1 unit is the minimum turning radius ρ of each AUV. For example, the turning radius of Iver2 AUV used in [8] is set to 5 m, so the proposed unit equals to 5 m. The number of targets varied from 10 to 50 in increments of 5, and the number of AUVs varied between 2 and 5. For the sake of simplicity, we choose the typical azimuth headings set for AUV movement as $\phi_{ij}\in \left\{\frac{b\pi }{4}\right\}$ with b=1,3,5,7. The proposed algorithm was implemented in Matlab and was developed on an Intel 2.5GHz i5-3210M CPU with 4GB RAM and running Windows 7. The computing time of the proposed algorithm with different targets number and AUV number are compared in Figure 6. The parameters for the GA is listed in Table 3.

**Figure 7:**
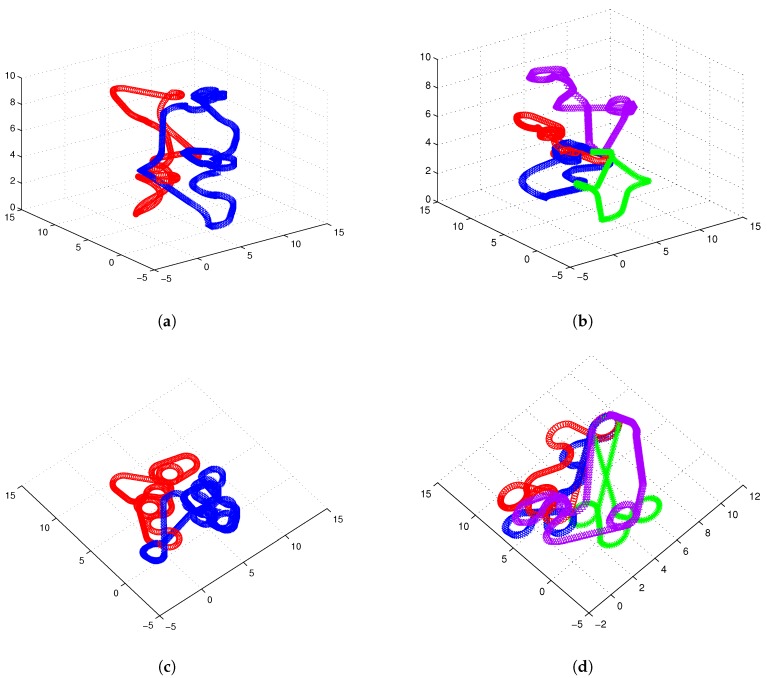
3D Dubins curves based targets tracking task assignment with 20 targets. Color online. (**a**) 3D Dubins curves with two AUVs; (**b**) 3D Dubins curve with four AUVs; (**c**) 3D Dubins curces projected 2D plane with two AUVs; (**d**) 3D Dubins curves projected 2D plane with four AUVs.

**Figure 8:**
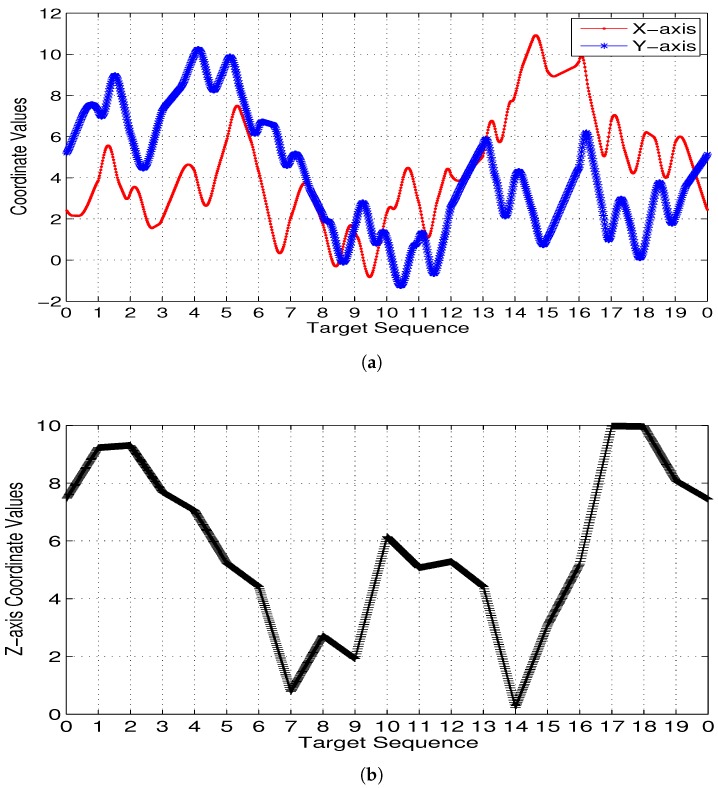
$G^{1}$ continuity of the 3D Dubins paths generated by the proposed algorithm. (**a**) in the X−Y plane; (**b**) in the *Z* axis.

**Figure 9:**
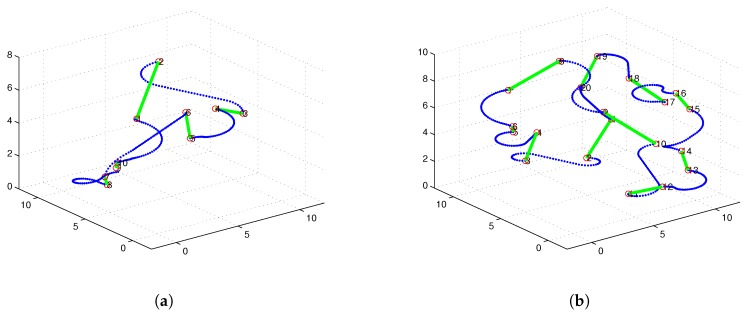
The 3D Alternating Algorithm (AA-3DTSP) with one AUV. (**a**) with 10 targets; (**b**) with 20 targets.

**Figure 10:**
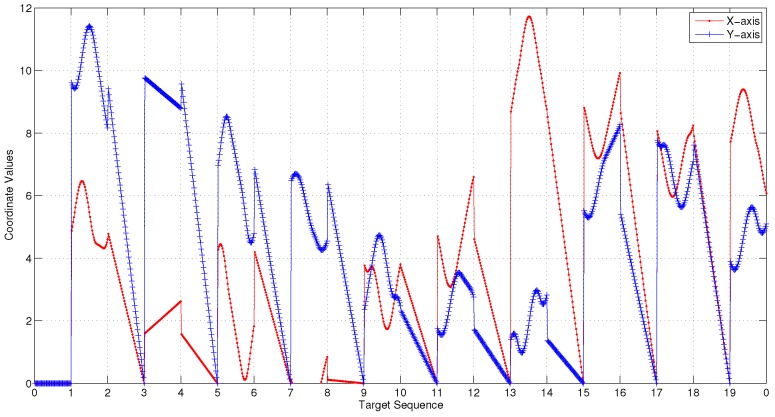
$G^{0}$ continuity in the X−Y coordinates for the AA-3DTSP algorithm. The *Z*-axis continuity is similar to that in Figure 8b.

Figure 7 illustrates 3D Dubins based MTSP trajectories of 2 or 4 AUVs generated by 3Dubins-MTSP algorithm. The x-axis and y-axis coordinates with their differential values are compared in Figure 8. In comparison, the 3D Alternating Algorithm (AA-3DTSP) extended the 2D Alternating Algorithm [23] by assigning *Z*-coordinates with linear interpolation and the resulting path is shown in Figure 9. A DTSP tour in (AA-3DTSP) can be constructed by retaining all odd-numbered edges (except the n-th), and replacing all even-numbered edges with minimum-length Dubins’ paths preserving the point ordering. In Figure 9, the Green curves denote odd-numbered edges, and the Blue curves denote even-numbered edges. It is clear non-smooth trajectories fail to $G^{1}$ continuity in either the *X*–*Y* plane nor the z-axis, as shown in Figure 10. Comparing Figure 10 to Figure 8, it is evident that cruise trajectories derived from our algorithm are $G^{1}$ continuous, however, cruise trajectories derived from Alternating Algorithm are only $G^{0}$ continuous because the tangent angle of each point on the path is not continuous. Therefore, 3D Alternating Algorithm (AA-3DTSP) is not appropriate for nonholonomic AUV and so it is excluded in the following simulations.

Next, we demonstrate the effectiveness of the THB-3Dubins-MTSP algorithm and TLB-3Dubins-MTSP constraints in comparison with the TSP without constraints and the Random Tour (RT) Algorithm. The Random Tour (RT) algorithm uses a set of random headings to achieve cruise paths without any constraints. The 3D Dubins based TSP (3Dubins-TSP) algorithm use only one AUV to trace all targets while cruising along 3D Dubins curves. Performance metrics include energy consumption, energy balance rate, cruise speed, and task life cycle. Energy consumption denotes the total energy consumption of all AUVs in the mission of tracking all targets, which is measured by the total tour length with assumption that each AUV consumes one unit energy at each unit tour length. Energy balance rate denotes with RMS (root mean square) value of energy consumption of each AUV, which is defined as Equation (28). Cruise speed is defined the maximal tour length of the AUV team in one cruise process, which denotes that the maximal time needed to finish one cruise for all targets. Task life cycle denotes the repeated number of each cruise mission, and it equals to the round number when one of AUV exhausts its energy. In this paper, it is assumed that the mobile AUV will carry out targets detection mission iteratively, and cruise lifetime is measured as the number of targets detection mission.

(28) $$RMS=\sqrt{\frac{\sum_{k=1}^{K}{\left(C\left(D_{k}\right)-\bar{C(D_{k})}\right)}^{2}}{K}}$$

**Figure 11:**
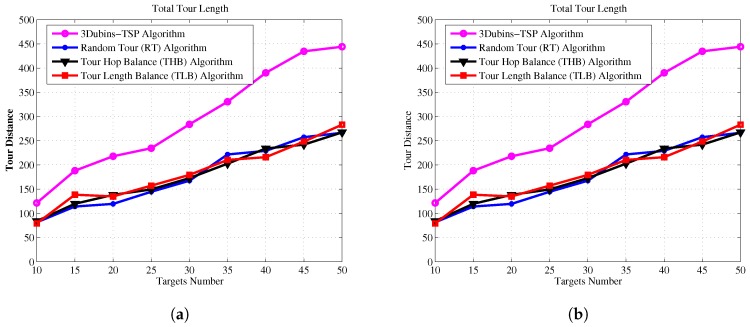
Energy consumption comparisons. (**a**) 2 AUVs; (**b**) 4 AUVs.

**Figure 12:**
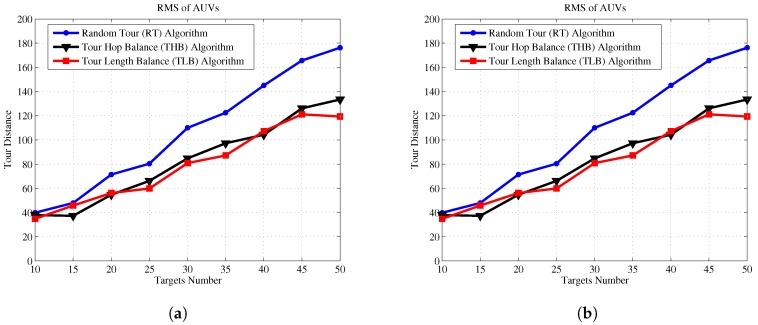
Energy balance comparisons. (**a**) 2 AUVs; (**b**) 4 AUVs.

**Figure 13:**
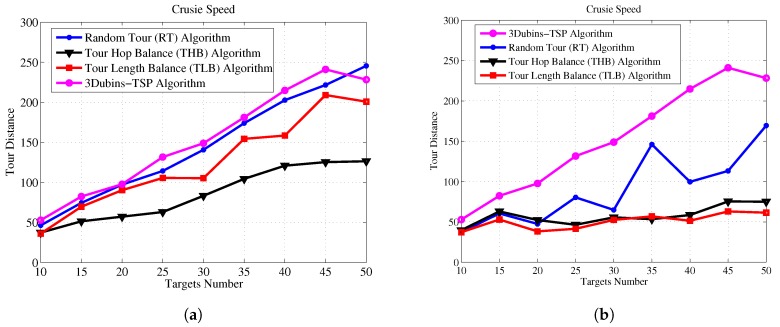
Cruise speed comparisons. (**a**) 2 AUVs; (**b**) 4 AUVs.

**Figure 14:**
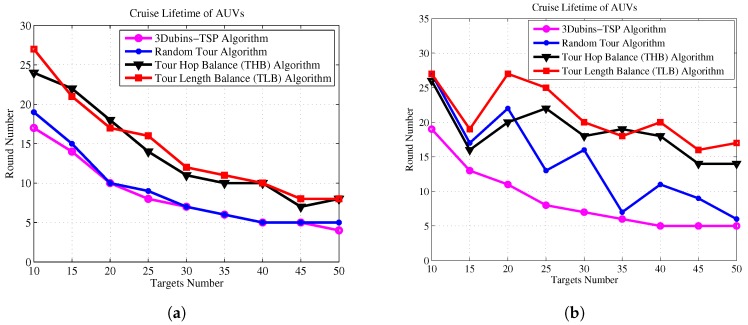
Task life cycle comparisons. (**a**) 2 AUVs; (**b**) 4 AUVs.

For a given number of targets, we simulated 100 Monte Carlo trails and computed the average length of 3D tours generated by different algorithms. The standard deviation value is relatively small since the proposed algorithms can achieve progressive optimization using a large number of iterations of GA. The comparison results of energy consumption, energy balance, cruise speed and task life cycle are shown in Figure 11, Figure 12, Figure 13 and Figure 14, respectively.

Figure 11 shows that our proposed algorithms consume almost the same energy comparing to Random Tour algorithm, but they are much lower than 3Dubins-TSP algorithm. Figure 12 shows the improvements of energy balance ratio, there is at most 50% improvement with Random Tour algorithm on the RMS metric. Figure 13 shows the cruise speed comparisons of different mechanism, we find the cruise distance (e.g., the maximal cruise time) will decrease with the proposed algorithms clearly, and it is even obvious with more AUVs. Figure 14 shows the task life cycle comparisons, it is obvious the lifetime can be extended with our proposed algorithms, especially with more targets in the underwater region. In summary, the proposed THB-3Dubins-MTSP and TLB-3Dubins-MTSP algorithms will improve performances such as energy balance ratio, cruise speed and task life cycle comparing to Random Tour (RT) algorithm greatly, thus verify that the proposed algorithms can achieve better performance with $G^{1}$ continuous constraints. Moreover, the proposed THB-3Dubins-MTSP and TLB-3Dubins-MTSP algorithms have similar performances.

## 5. Conclusions and Future Work

This paper has studied the 3D Dubins curves for target assignment and path planning for multiple underwater targets visited by multiple AUVs. The MTSP for 3D path planning is solved by using the inverse of the Euclidean distances as the fitness function and the Tour Hop Balance (THB) and Tour Length Balance (TLB) constraints as the stop criterion. The resulting target assignment (tour sequence) is then projected onto the 2D X−Y planes and 2D Dubins curves are designed with a set of possible headings and with nonholonomic motion constraints. The resulting 2D Dubins curves are interpolated linearly to obtain the *Z*-coordinates of each 2D curve. We derived the path length calculation for 3D Dubins curves and analyzed the $G^{1}$ continuity of the 3D Dubins curve. It is demonstrated that the linear interpolation fails to achieve $G^{1}$ continuity in the *Z* coordinate, and other smooth interpolation may have to be used when the continuity is required. Moreover, we find there are small collision probabilities between AUVs from above analysis. Therefore, in our future work, we will study how to avoid multiple underwater obstacles and inter-AUV collision for path planning of multi-AUV team.

## Figures and Tables

**Table 1:** List of Notations.

Notation	Definition
T	the set of static targets
A	the set of mobile AUVs
$T_{n}$	the *n*-th static target
$A_{k}$	the *k*-th mobile AUV
*N*	total number of static targets
*K*	total number of mobile AUVs
$D_{k}$	tour sequence for the *k*th AUV, k=1,2,…,K
$S_{k}$	tour trajectory for the *k*th AUV, k=1,2,…,K
$N_{k}$	the number of targets in sequence $D_{k}$
$\mu (N_{k})$	intra-AUV mean of the number of assigned targets
$L_{k}$	tour length of sequence $D_{k}$
$\mu (L_{k})$	intra-AUV mean of the assigned tour lengths
x=(x,y)	point coordinates in 2D plane
ϕ	heading in 2D Dubins curve
$L_{2D}$	length of 2D Dubins curve
$L(\left[x_{0},\phi_{0}\right];\left[x_{1},\phi_{1}\right])$	length of 2D Dubins curve from point $x_{0}$ to point $x_{1}$
X=(x,y,z)	point coordinates in 3D space
Φ=(ϕ,θ)	azimuth and elevation headings of 3D Dubins curve
$L_{3D}$	length of 3D Dubins curve
$L(\left[X_{0},\Phi_{0}\right];\left[X_{1},\Phi_{1}\right])$	length of 3D Dubins curve from point $X_{0}$ to point $X_{1}$
Λ	the set of possible headings in 2D plane
*ℜ*	set of Dubins curves
t,p,q	lengths of three segments of a Dubins curve
$I_{n,k}^{u}$	binary indicator

**Table 2:** Decision table for shortest 2D Dubins curves based on the quadrant numbers of the starting and ending angles.

$a_{\mathrm{ij}}$	j=1	j=2	j=3	j=4
*i* = 1	RSL	RSR/RSL	RSR/LSR	LSR/RSL/RSR
*i* = 2	LSL/RSL	LSL/RSL/RSR	RSR	RSR/RSL
*i* = 3	LSL/LSR	LSL	RSR/LSR/LSL	RSR/LSR
*i* = 4	RSL/LSR/LSL	LSL/RSL	LSL/LSR	LSR

**Table 3:** The GA Parameters for the MTSP.

Parameters	Values
Population Size	80
Maximal Iterations	5000
Mutation Ratio	5%
Group Size	5
